# Integration of Entomopathogenic Fungi into IPM Programs: Studies Involving Weevils (Coleoptera: Curculionoidea) Affecting Horticultural Crops

**DOI:** 10.3390/insects11100659

**Published:** 2020-09-25

**Authors:** Kim Khuy Khun, Bree A. L. Wilson, Mark M. Stevens, Ruth K. Huwer, Gavin J. Ash

**Affiliations:** 1 Faculty of Agronomy, Royal University of Agriculture, P.O. Box 2696, Dangkor District, Phnom Penh, Cambodia; 2 Centre for Crop Health, Institute for Life Sciences and the Environment, University of Southern Queensland, Toowoomba, Queensland 4350, Australia; bree.wilson@usq.edu.au (B.A.L.W.); gavin.ash@usq.edu.au (G.J.A.); 3 NSW Department of Primary Industries, Yanco Agricultural Institute, Yanco, New South Wales 2703, Australia; mark.stevens@dpi.nsw.gov.au; 4 Graham Centre for Agricultural Innovation (NSW Department of Primary Industries and Charles Sturt University), Wagga Wagga, New South Wales 2650, Australia; 5 NSW Department of Primary Industries, Wollongbar Primary Industries Institute, Wollongbar, New South Wales 2477, Australia; ruth.huwer@dpi.nsw.gov.au

**Keywords:** attract-and-kill, *Bacillus thuringiensis*, *Beauveria*, endophyte, entomopathogenic nematode, *Metarhizium*, repellent volatile, sterile male, transmission, weevil

## Abstract

**Simple Summary:**

Horticultural crops are vulnerable to attack by many different weevil species. Fungal entomopathogens provide an attractive alternative to synthetic insecticides for weevil control because they pose a lesser risk to human health and the environment. This review summarises the available data on the performance of these entomopathogens when used against weevils in horticultural crops. We integrate these data with information on weevil biology, grouping species based on how their developmental stages utilise habitats in or on their hostplants, or in the soil. These patterns of habitat usage can help identify the stages during which pest species are at their most vulnerable, and also help to determine the most effective ways to deploy entomopathogens for their control.

**Abstract:**

Weevils are significant pests of horticultural crops and are largely managed with insecticides. In response to concerns about negative impacts of synthetic insecticides on humans and the environment, entomopathogenic fungi (EPF) have been developed as an alternative method of control, and as such appear to be “ready-made” components of integrated pest management (IPM) programs. As the success of pest control requires a thorough knowledge of the biology of the pests, this review summarises our current knowledge of weevil biology on nut trees, fruit crops, plant storage roots, and palm trees. In addition, three groups of life cycles are defined based on weevil developmental habitats, and together with information from studies of EPF activity on these groups, we discuss the tactics for integrating EPF into IPM programs. Finally, we highlight the gaps in the research required to optimise the performance of EPF and provide recommendations for the improvement of EPF efficacy for the management of key weevils of horticultural crops.

## 1. Introduction

Insect pests are one of the main constraints to global crop production and reduce crop yields by 30–40%, equating to US$300 to 470 billion worth of production losses each year [1,2]. To manage them, synthetic insecticides are routinely used by commercial growers, with at least US$16 to 20 billion being spent on insecticides annually [1,3]. However, a sole reliance on insecticides is not considered sustainable as they are often harmful to endemic natural enemies within crops and may induce insecticide resistance in the target pest [4]. The same insecticides may also increase the frequency of primary and secondary pest outbreaks [5]. For example, pecan scab (*Venturia effusa*), pecan weevil (*Curculio caryae*) and pentatomid stink bugs (*Nezara viridula* and *Euschistus* sp.) are the key problems in pecan (*Carya illinoinensis*) plantations and to minimise their impact, preventive applications of broad-spectrum pesticides are used. Pyrethroids and carbaryl are used for control of late-season pecan weevil and kernel-feeding hemipterans; however, these insecticides also destroy aphidophagous insects and repel or kill predatory mites. Consequently, aphid and phytophagous mite resurgences are often observed [6]. Fungicides can also contribute to pest outbreaks and resurgence as a consequence of their impact on entomopathogenic fungi (EPF) [6,7]. The fungicides applied to control pecan scab are reported to kill the EPF that provide control of pecan aphids [8] and as a consequence, secondary outbreaks of aphids require additional application of insecticides [6].

Microbial biopesticides have been recognised as alternatives to synthetic insecticides, since they can have minimal impacts on non-target organisms, prevent pesticide resistance, and are less toxic to both humans and the environment [9,10]. On a global scale, microbial biopesticides account for approximately US$3.3 billion or around 8% of all pesticides sold [11], but they have long-term potential for increased usage over the next few decades [12]. Among the microbial biopesticides, EPF are the second-highest selling, accounting for around 9% of all microbial biopesticides sold globally [11]. Their popularity stems from their potential to control a wide range of insect pests [13,14] and their suitability for organic and sustainable crop production [15]. In addition to their direct impact on insect pests, EPF have also been reported to act as endophytes within host plants [16], can be integrated with attractants for attract-and-kill pest management approaches [17,18], and can be combined with sterile males for integration with the Sterile Insect Technique (SIT) [19,20,21]. Entomopathogenic fungi may also have synergistic interactions with some beneficial arthropods (predators, parasitoids, pollinators) [22,23,24], other entomopathogens (bacteria and nematodes) [25,26] and synthetic insecticides [27,28] that could be exploited within IPM programs on various crops.

Weevils are amongst the most important pests of horticultural crops. They often have behaviours and habitats that can make some insecticides difficult to deploy. For example, pecan weevil is a major pest of pecans in the southern United States [29] where the damage caused by larvae and adults can reduce yields by up to 80% [30]. This weevil has a lengthy and complicated life cycle (90% of the population complete their life cycle in 2 years while 10% take up to 3 years) with the larvae and pupae occurring inside nuts and in the soil, respectively [29]. This makes the opportunities to control this weevil using contact insecticides limited to only the adults.

Another important weevil in horticulture is the coffee berry borer (*Hypothenemus hampei*). It is a major pest of coffee (*Coffea arabica* and *C. canephora*) worldwide [31], with the damage caused by the larvae and adults estimated to cost the industry around US$500 million annually [32]. This borer is also difficult to control as the larval and pupal stages only occur inside the coffee berries [31]. There are many other weevil species in which one or more developmental stages live within the host plant and/or the soil and cause significant damage to horticultural crops (Table 1).

## 2. Methodology

**Figure 1:**
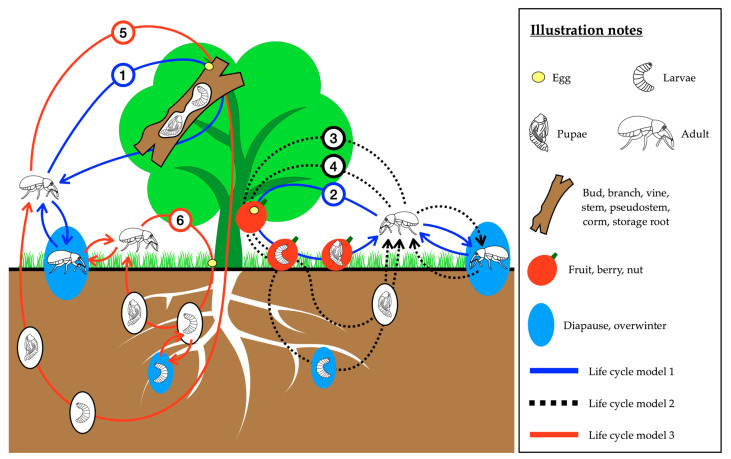
Overview of the life cycles of weevils attacking horticultural crops. The weevil species which were classified into subcategories 1–6 and life cycle models 1–3 in this figure are listed in Table 3.

This review describes the potential use of EPF (particularly *Beauveria* spp. and *Metarhizium* spp.) alone or in combination with other management techniques to control weevils in horticultural crops. Studies using *Metarhizium* spp. or *Beauveria* spp. for managing weevils affecting horticultural crops were identified using databases including Web of Science (http://www.webofknowledge.com), SCOPUS (https://www.scopus.com), CAB Abstracts (https://www.cabdirect.org) and Google Scholar (https://scholar.google.com). A total of 1666 articles (Figure S1) were identified from searches using the terms “*Metarhizium*”, “*Beauveria*”, “Weevil”, and “Curculionidae”. After removing duplicates, 566 article titles were screened and 391 were excluded from this review because they fell outside the scope of this review due to the host crop involved, or other factors. The final 175 full-text articles, which show a strong bias towards perrenial crops, are included in this review. Fourty-four weevil species were identified as having a major impact on horticultural crops (Table 1). Studies published between 1973 and 2020 that dealt with the use of EPF for weevil control involved 26 of these species, and of these, data on life cycle duration was available for 21 (Table 2, Figure S2). The 26 weevil species used in experiments involving EPF could be grouped according to their patterns of habitat utilisation throughout their life cycles (Figure 1, Table 3). Successful pest management requires a thorough knowledge of pest biology [33], and in this review we combine published data with these patterns of habitat utilisation to identify the optimal approaches for integrating EPF into weevil IPM programs, targeting the most vulnerable developmental stages for each weevil group.

In this review, weevils are defined as the superfamily Curculionoidea, following the taxonomy of Oberprieler et al. [34] where the Curculioninae, Cyclominae, Dryophthorinae, Entiminae, Molytinae, and Scolytinae have subfamily status within the Curculionidae, and the Brentinae are a subfamily within the Brentidae.

**Table 1:** Important weevil species of horticultural crops, including crops impacted, geographical distribution and economic impact.

Weevil Species	Common Name	Family: Subfamily ^1^	Distribution ^2^	Crops	Damaging Stages ^3^	Economic Impact ^4^	Ref.
*Aclees* sp. cf. *foveatus* (Voss)	Fig weevil	Cur: Mol	IT	Fig	A & L	n/a	[35]
*Aegorhinus superciliosus* (Guérin)	Raspberry weevil	Cur: Cyc	AR & CL	Blueberries, raspberries, strawberry	A & L	n/a	[36,37]
*Anthonomus musculus* (Say)	Cranberry weevil	Cur: Cur	North-Eastern US & CA	Blueberries, cranberries	A & L	n/a	[38]
*Anthonomus piri* (Kollar)	Apple bud weevil	Cur: Cur	EUR & GB	Apple, pears	A & L	n/a	[39]
*Anthonomus pomorum* (L.)	Apple blossom weevil	Cur: Cur	EUR	Apple, pears	A & L	n/a	[39]
*Anthonomus rubi* (Herbst)	Strawberry blossom weevil	Cur: Cur	EUR & GB	Strawberry, blackberry, raspberry	A & L	MCL between 36–90%	[40,41]
*Anthonomus signatus* (Say)	Strawberry bud weevil	Cur: Cur	US & CA	Strawberry	A & L	MCL up to 100% in New York & 70% in Quebec	[42]
*Blosyrus asellus* (Olivier)	Rough sweetpotato weevil	Cur: Ent	US	Sweetpotato	A & L	n/a	[43]
*Conotrachelus nenuphar* (Herbst)	Plum curculio	Cur: Mol	Eastern & central NAM (US, CA)	Pome & stone fruits	A & L	MCL up to 85% in unsprayed orchard	[44,45]
*Conotrachelus psidii* (Marshall)	Guava weevil	Cur: Mol	BO, BR, CO, MX, PY & VE	Guava	L	MCL up to 100% in Rio de Janeiro, Brazil	[46,47]
*Cosmopolites sordidus* (Germar)	Banana weevil	Cur: Dry	Tropical regions worldwide	Banana & plantain	A & L	MCL up to 50%	[31,48]
*Curculio caryae* (Horn)	Pecan weevil	Cur: Cur	Southern US	Pecan	A & L	MCL between 30–80%	[29,30]
*Curculio caryatrypes (*Boheman)	Larger chestnut weevil	Cur: Cur	Central-eastern US	Chestnut	A & L	n/a	[49]
*Curculio elephas* (Gyllenhal)	Chestnut weevil	Cur: Cur	Central & Southern EUR, North AFR	Chestnut	A & L	MCL up to 90% in Italy	[39,50]
*Curculio nucum* (L.)	Hazelnut weevil	Cur: Cur	PAL, also present in North AFR	Hazelnut	A & L	MCL up to 80% in the unprotected orchards in Spain	[51,52]
*Curculio sayi* (Gyllenhal)	Lesser chestnut weevil	Cur: Cur	Central-eastern US	Chestnut	A & L	n/a	[49]
*Curculio sikkimensis* (Heller)	Chestnut weevil	Cur: Cur	CN, IN, JP & KR	Chestnut	A & L	n/a	[53,54]
*Cylas formicarius* (F.)	Sweetpotato weevil	Bre: Bre	Tropical regions worldwide	Sweetpotato	A & L	MCL up to 100%	[31,55]
*Cylas puncticollis (*Boheman), *C. brunneus* (F.)	African sweetpotato weevil	Bre: Bre	AFR (sub-Saharan)	Sweetpotato	A & L	MCL up to 97%	[55,56]
*Diaprepes abbreviatus* (L.)	Citrus root weevil	Cur: Ent	US & several CAR	Citrus, sugarcane	L	n/a	[57]
*Heilipus lauri* (Boheman)	Avocado seed weevil	Cur: Mol	CO & MX	Avocado	A & L	MCL between 60–70% in Mexico	[58,59]
*Hypothenemus hampei* (Ferrari)	Coffee berry borer	Cur: Sco	AFR, ASI, OCE, SCA & US	Coffee	A & L	MCL between 40 - 90%. EAL around US$215–358 million in Brazil or around US$500 million worldwide	[31,32,60]
*Kuschelorhynchus macadamiae* (Jennings & Oberprieler)	Macadamia seed weevil	Cur: Cur	Eastern AU	Macadamia	A & L	MCL up to 15%	[61,62]
*Odoiporus longicollis* (Olivier)	Banana stem weevil	Cur: Dry	Tropical ASI	Banana & plantain	A & L	MCL between 10–90%	[63]
*Otiorhynchus clavipes* (Bonsdorff)	Red-legged weevil	Cur: Ent	Western EUR	Plum, apple, berry crops, grapevine	A & L	n/a	[39]
*Otiorhynchus ovatus* (L.)	Strawberry weevil	Cur: Ent	EUR & NAM	Strawberry, berry crops	A & L	MCL up to 100% in Saxony, Germany	[39]
*Otiorhynchus rugifrons (*Gyllenhal)	Strawberry root weevil	Cur: Ent	EUR	Strawberry	A & L	n/a	[39]
*Otiorhynchus rugosostriatus* (Goeze)	Rough strawberry root weevil	Cur: Ent	EUR, NAM & MED	Strawberry, berry crops	A & L	n/a	[39]
*Otiorhynchus singularis* (L.)	Clay-coloured weevil	Cur: Ent	EUR & NAM	Apple, pear, berry crops, grapevine	A & L	n/a	[39]
*Otiorhynchus sulcatus* (F.)	Black vine weevil	Cur: Ent	EUR, NAM & AUA	Grapevines, berry crops	A & L	n/a	[39,64]
*Pantorhytes plutus* (Oberthür)	Cacao weevil	Cur: Ent	PG	Cacao	L	n/a	[65,66]
*Phlyctinus callosus* (Schönherr)	Banded fruit weevil	Cur: Ent	AU, NZ & ZA	Grapevines, pome fruit, stone fruits	A & L	MCL up to 40%	[67,68]
*Pityophthorus juglandis* (Blackman)	Walnut twig beetle	Cur: Sco	south-western US & MX	Walnut	A & L	n/a	[69]
*Rhynchophorus bilineatus* (Montrouzier)	Black palm weevil	Cur: Dry	ID, PG & SB	Palm	L	n/a	[70]
*Rhynchophorus cruentatus* (F.)	Palmetto weevil	Cur: Dry	Florida & south-eastern US	Palm	L	n/a	[70]
*Rhynchophorus ferrugineus* (Olivier)	Red palm weevil	Cur: Dry	ASI, AU & MED	Palm	L	EAL around US$5–26 million in the Middle East	[71,72]
*Rhynchophorus palmarum* (L.)	American palm weevil	Cur: Dry	MX & SCA	Palm	L	MCL up to 15%	[70,73]
*Rhynchophorus phoenicis* (F.)	African palm weevil	Cur: Dry	AFR	Palm	L	n/a	[70]
*Rhynchophorus quadrangulus* (Queden)	n/a	Cur: Dry	AFR	Palm	L	n/a	[70]
*Scolytus amygdali* (Guérin-Méneville)	Almond bark beetle	Cur: Sco	MED	Almond, apricot, peach	A & L	n/a	[39]
*Scolytus mali* (Bechstein & Scharfenberg)	Large fruit bark beetle	Cur: Sco	EUR & PAL	Apple, plum, pear	A & L	n/a	[39]
*Scolytus rugulosus* (Müller)	Fruit bark beetle	Cur: Sco	EUR	Apple, pear, plum	A & L	n/a	[39]
*Xyleborus affinis* (Eichhoff)	Ambrosia beetle	Cur: Sco	MX & US	Avocado, mango, macadamia, walnut	A & L	n/a	[74,75,76]

^1^ Bre: Bre = Brentidae: Brentinae, Cur: Cur = Curculionidae: Curculioninae, Cur: Cyc = Curculionidae: Cyclominae, Cur: Dry = Curculionidae: Dryophthorinae, Cur: Ent = Curculionidae: Entiminae, Cur: Mol = Curculionidae: Molytinae, Cur: Sco = Curculionidae: Scolytinae. ^2^ AFR = Africa, AR = Argentina, ASI = Asia, AU = Australia, AUA = Australasia, BO = Bolivia, BR = Brazil, CA = Canada, CAR = Caribbean nations, CL = Chile, CN = China, CO = Colombia, EUR = Europe, GB = United Kingdom, ID = Indonesia, IN = India, IT = Italy, JP = Japan, KR = Korea, MED = Mediterranean area, MX = Mexico, NAM = North America, NZ = New Zealand, OCE = Oceania, PAL = Palaearctic, PG = Papua New Guinea, PY = Paraguay, SCA = South and Central America, SB = Solomon Islands, US = United States, VE = Venezuela, ZA = South Africa (code follows https://www.iso.org/). ^3^ A = adults, L = Larvae. ^4^ n/a = Specific data not available, MCL = may cause yield or crop loss, EAL = estimated annual loss. Ref. = References.

**Table 2:** Weevil life stage durations (where known) for important horticultural pest species used in experiments with EPF.

Weevil Species	Egg (Days)	Larvae (Days)	Pupae (Days)	Adult (Days)	Generation	Ref.
*Aclees* sp. cf. *foveatus*	10–20	n/a	n/a	n/a	2 generations/year	[35]
*Anthonomus signatus*	6–14	21–28	5–8	n/a	32–64 days/generation, 1 generation/year	[42,77]
*Conotrachelus nenuphar*	2–12	14–21	30	n/a	57 days/generation	[78]
*Conotrachelus psidii*	2–6	8–27	14–18	<418	108–280 days/generation	[79]
*Cosmopolites sordidus*	5–8	14–21	5–7	<730	1–6 months/generation	[31,80]
*Curculio caryae*	n/a	30	270–1080	n/a	2–3 years/generation	[29,81]
*Curculio elephas*	n/a	730–1095	90–150	n/a	1 generation/year in Italy	[50,82]
*Curculio nucum*	>7	28–35	< 365	90	1 generation/year in Turkey	[39,83]
*Cylas formicarius*	3–7	7–11	5–7	<240	5–8 generations/year in United States	[31,55]
*Cylas puncticollis*	< 5	<23	<14	<141	20–25 days/generation	[56]
*Diaprepes abbreviatus*	7–10	240–450	15–30	<147	5–18 months/generation	[84]
*Heilipus lauri*	<13	<49	<15	n/a	76 days/generation	[58]
*Hypothenemus hampei*	5–9	10–26	4–9	<157	25–35 days/generation, >8 generations/year in African countries, 2–3 generations/year in Colombia	[31,85]
*Kuschelorhynchus macadamiae*	6	28	4	n/a	At least 3 generations/year	[86]
*Odoiporus longicollis*	3–8	30–60	17–22	50–95	53–95 days/generation	[87]
*Otiorhynchus sulcatus*	>8	84–211	10–50	n/a	1 generation/year	[64]
*Pantorhytes plutus*	n/a	90–270	14	365–730	4–11 months/generation	[88]
*Phlyctinus callosus*	6–15	n/a	7–21	n/a	1–2 generations/year	[89]
*Pityophthorus juglandis*	n/a	n/a	n/a	n/a	7 weeks/generation, 2 generations/year	[90]
*Rhynchophorus ferrugineus*	1–6	25–105	11–45	n/a	45 days/generation in the Philippines, 139 days/generation in Spain; 3–4 generations/year in India, up to 21 generations/year in Egypt	[71]
*Scolytus amygdali*	n/a	n/a	n/a	n/a	>3 generations/year in the Mediterranean area	[91]

Note: *Aegorhinus superciliosus*, *Blosyrus asellus*, *Curculio sikkimensis*, *Rhynchophorus bilineatus* and *Xyleborus affinis* were not included in this table as specific data are not available. n/a = specific data not available. Ref. = References.

**Table 3:** Major horticultural weevil species used in experiments with EPF grouped according to their different life cycle habitat utilisation models. Subcategories 1–6 correspond to those shown in Figure 1.

Life Cycle Model	Subcategory	Weevil Species	Common Name
**Model 1: Larvae and Pupae in the Host Plant**	**1**	*Aclees* sp. cf. *foveatus*	Fig weevil
		*Anthonomus signatus*	Strawberry bud weevil
		*Blosyrus asellus*	Rough sweetpotato weevil
		*Cosmopolites sordidus*	Banana weevil
		*Cylas formicarius*	Sweetpotato weevil
		*Cylas puncticollis*	African Sweetpotato weevil
		*Odoiporus longicollis*	Banana stem weevil
		*Pantorhytes plutus*	Cacao weevil
		*Pityophthorus juglandis*	Walnut twig beetle
		*Rhynchophorus ferrugineus*	Red palm weevil
		*Rhynchophorus bilineatus*	Black palm weevil
		*Scolytus amygdali*	Almond bark beetle
		*Xyleborus affinis*	Ambrosia beetle
	**2**	*Heilipus lauri*	Avocado seed weevil
		*Hypothenemus hampei*	Coffee berry borer
		*Kuschelorhynchus macadamiae*	Macadamia seed weevil
**Model 2: Larvae in the Host Plant and Pupae under the Ground**	**3**	*Curculio caryae*	Pecan weevil
		*Curculio elephas*	Chestnut weevil
		*Curculio sikkimensis*	Chestnut weevil
		*Curculio nucum*	Hazelnut weevil
	**4**	*Conotrachelus nenuphar*	Plum curculio
		*Conotrachelus psidii*	Guava weevil
**Model 3: Larvae and Pupae under the Ground**	**5**	*Diaprepes abbreviatus*	Citrus root weevil
	**6**	*Aegorhinus superciliosus*	Raspberry weevil
		*Otiorhynchus sulcatus*	Black vine weevil
		*Phlyctinus callosus*	Banded fruit weevil

## 3. Life Cycle Patterns of Weevils Affecting Horticultural Crops

The three patterns of weevil life cycles which occur in association with horticultural crops are summarised in Figure 1 and Table 3. Adult weevils are normally active on the host plant during feeding and mating. Three locations on or around the host plant are potentially suitable sites for weevils to lay eggs, depending on the species’ biology; (1) in/on the fruit, berry or nut; (2) in/on the bud, leaf, branch, vine, stem, pseudostem, corm, or storage root and (3) in the soil or at the base of the plant. As larvae hatch from the eggs they move to, or are already positioned at the location of the larval food source; (1) in the bud, branch, stem, pseudostem, corm, storage root or root; (2) in the fruit, berry or nut. The larval and pupal habitats never leave the immature stages exposed where they could be directly sprayed with either entomopathogens or contact insecticides. The mature larvae pupate either (1) in the berry, nut, bud, branch, vine, stem, pseudostem, corm or storage root of the host plant, or (2) under the ground. Some species need to diapause or overwinter in the soil as either larvae (pecan weevil, chestnut weevil, hazelnut weevil, black vine weevil, banded fruit weevil) or adults (walnut twig beetle, strawberry bud weevil, macadamia seed weevil, chestnut weevil, hazelnut weevil, black vine weevil). After days to months (Table 2), the adults emerge from the host plant or the ground and establish the next generation. Studies on the impacts of EPF on weevils are grouped together based on these life cycle models and discussed in the following sections of this review. Model 1: larvae and pupae both in the host plant; Model 2: larvae in the host plant and pupae under the ground; Model 3: larvae and pupae both under the ground (Table 3).

## 4. Effect of Fungal Entomopathogens on Weevils with Life Cycle Model 1: Larvae and Pupae in the Host Plant

In total, 61 screening studies have demonstrated the efficacy of *Metarhizium* spp. and *Beauveria* spp. on weevils with Model 1 habitat utilisation. The aim of these studies was to find the most effective isolate of each EPF through the evaluation of either commercial strains/products or local isolates. In total, 98 isolates of *Metarhizium* spp., 275 isolates of *Beauveria* spp. and 16 commercial strains/products were used for the bioassays. Of the 61 published papers, 55 examined the effects of EPF using aqueous conidial suspensions and only 6 papers examined efficacy using dried conidia applied to different substrates. For bioassays with aqueous conidia, adults or larvae of *Aclees* sp. cf. *foveatus* [92], *Anthonomus signatus* [93], *Cosmopolites sordidus* [94,95,96,97,98,99,100,101,102], *Cylas formicarius* [103,104], *C. puncticollis* [105], *Heilipus lauri* [106], *Hypothenemus hampei* [107,108,109,110,111,112,113,114,115,116,117,118], *Kuschelorhynchus macadamiae* [119], *Odoiporus longicollis* [120,121,122], *Pantorhytes plutus* [65], *Pityophthorus juglandis* [123], *Rhynchophorus ferrugineus* [124,125,126,127,128,129,130,131,132,133,134,135,136,137,138,139,140,141,142,143], *Scolytus amygdali* [144] and *Xyleborus affinis* [76] were immersed for 3–90 s or sprayed with conidial suspensions at varying concentrations. For the studies with dried conidia, *C. sordidus* [145,146] and *R. ferrugineus* [147,148,149,150] were rolled in (5 min) or allowed to walk on dried conidia on substrates such as fungal media, rice or wheat (15 min to 24 h).

Efficacy comparisons between EPF species showed that *B. bassiana* performed better than *M. anisopliae* in killing *Aclees* sp. cf. *foveatus* [92], *C. sordidus* [145], *H. hampei* [111] and *H. lauri* [106], whilst the opposite result was found with *R. ferrugineus* [126,147,148]. Other studies showed that *M. anisopliae* and *B. bassiana* were equally effective in killing *C. sordidus* [95,97,102], *C. puncticollis* [105], *K. macadamiae* [119], *P. juglandis* [123] and *R. ferrugineus* [129,134,143]. The majority of highly virulent isolates and/or the highest conidial concentrations tested in each study resulted in moderate (60–80%) to high levels (>80%) of mortality in the target weevils, except for *C. sordidus* [98,100], *H. hampei* [115] and *X. affinis* [76] where only a low level of mortality was obtained. This could be the result of poor virulence of the fungal isolates since the number of conidia used in these studies was high (adults immersed in 10^8^ conidia mL^−1^ [98,100] or spray application at 10^7^–10^8^ conidia mL^−1^ [76,115]). Two studies showed that using biosynthesised silver nanoparticles for disseminating *M. anisopliae* or *B. bassiana* could improve efficacy against *R. ferrugineus* by 10% in comparison to traditional spray applications of conidial suspensions [136,140]. Formulated EPF [92,119,124,127] and non-formulated commercial strains [107,108,123] provided strong and consistent control of weevils with Model 1 habitat patterns under laboratory conditions. For example, Abdel-Samad et al. [124] and Hajjar et al. [127] found that Agronova^®^ and Broadband^®^ products which contain *B. bassiana* in an oil formulation caused 86–100% mortality of *R. ferrugineus* when the recommended rate of 10^9^ conidia mL^−1^ was used to treat adults. Castrillo et al. [123] reported that *B. bassiana* strain GHA and *M. brunneum* strain F52 at 10^6^ conidia mL^−1^ were highly virulent and caused high mortality (>90%) of *P. juglandis* 5 days after treatment.

Although the majority of these strains induced moderate (60–80%) to high (>80%) levels of mortality to weevils in controlled environments, economic control of using EPF in the field may not be achieved as readily. As EPF can take at least 15 days to cause weevil mortality of more than 80% under field conditions, the targeted weevils are likely to cause at least some damage to the crops either by feeding or laying viable eggs during the intervening period. Twenty-two studies have evaluated the efficacy of EPF on weevils of walnuts, almonds, bananas, coffee, strawberries, sweetpotatoes and palms in the glasshouse or under field conditions. Of these, fourteen examined spray application of EPF onto the plant and four evaluated either the injection of EPF into the space between the stem and petiole insertion point, or application of the dried formulated fungi onto the plant crown before or after weevil establishment. Only four papers discussed the natural occurrence and prevalence of EPF in the field, and these papers involved the control of either *C. sordidus* [151], *H. hampei* [152,153] or *R. ferrugineus* [154]. The stem injection technique was only applied against *R. ferrugineus* larvae and pupae, which remain inside the plant [131,132,155,156], while the spray applications were invariably targeting adult weevils such as *A. signatus* [157], *C. sordidus* [97,158], *Cylas* spp. [159], *H. hampei* [109,160,161,162,163], *P. juglandis* [123], *R. ferrugineus* [125,164,165] and *S. amygdali* [144], which are exposed outside the plant as adults. Variable control was achieved according to the fungal species used [160], fungal persistence [155,157], application technique [125], frequency of the application [163], weather conditions [109,151,152,153] and insect species. Overall, spray application of the most virulent isolates caused low (<60%) to moderate (60–80%) levels of mortality to the target weevils, whilst application by stem injection led to moderate to high (>80%) mortality of *R. ferrugineus* larvae and pupae.

Eight studies evaluated the effects of incorporating conidia within or on top of topsoil and plant growing media (compost, sawdust) on the mortality of weevils with Model 1 habitat utilisation. These studies aimed to use EPF to control weevils moving through or across the topsoil, plant growing media or across infective fungal substrates used to create a protective barrier around host plants. Of the eight papers, three examined mortality under laboratory conditions and five assessed efficacy in the glasshouse or field. In the laboratory, EPF were sprayed or applied to the soil or growing media before introducing the weevils. High mortality (>80%) of the weevils (*C. formicarius* and *R. ferrugineus*) occurred [126,166], except in the study by Francardi et al. [149]. The low mortality (12–20%) of *R. ferrugineus* recorded by Francardi et al. [149] could be the result of there being insufficient conidia in the soil to adhere to and infect the adults. This was confirmed by the same authors who replaced soil with conidiated rice for adults to move across, improving the mortality rate to more than 85% [149]. However, only low (<60%) to moderate (60–80%) mortality of weevils was achieved in the field when this treatment method was used (24% to 63%, 8% to 75% and 25 to 62% for *C. sordidus* [167,168], *H. hampei* [113,169] and *P. juglandis* [90], respectively). This may have been a consequence of insufficient amounts of conidia being applied, the long period between fungal application and the emergence of adult weevil populations, and/or the effect of the unstable microclimate near the topsoil where there would have been large fluctuations in temperature and humidity. This issue is discussed further in the section on weevils with Model 2 and 3 habitat utilisations.

In order to improve weevil management, combinations of EPF with other biological control agents (BCAs) and attractants have also been explored. Three studies evaluated synergistic interactions of EPF with entomopathogenic nematodes (EPNs) and *Bacillus thuringiensis* against *R. ferrugineus* [170,171,172]. Saleh et al. [171] reported that *B. bassiana* behaved synergistically with *Steinernema carpocapsae* (EPNs) and killed *R. ferrugineus* adults in just 24 h when both BCAs were co-applied. In contrast, Wakil et al. [172] reported that EPF and EPNs could not be applied at the same time. They found that 72–89% mortality of *R. ferrugineus* larvae could be obtained when EPNs (*Heterorhabditis bacteriophora*) were applied two weeks after *B. bassiana* or *M. anisopliae*. However, low mortality (below 30%) was observed when EPF or EPNs were applied alone, or 45–61% mortality when EPF and EPNs were applied simultaneously, supporting the synergy between EPF and EPNs when used with appropriate timings [172]. Malik et al. [170] reported that *B. thuringiensis* behaved synergistically with *B. bassiana* for managing *R. ferrugineus.* When both entomopathogens were co-applied on *R. ferrugineus* larvae they caused substantially more mortality and reduced the percentages of pupation and adult emergence than *B. bassiana* or *B. thuringiensis* used alone [170].

Combinations of EPF with attractants such as a methanol/ethanol mixture, aggregation pheromones or sex pheromones were tested against *C. sordidus* [173,174,175], *C. formicarius* [176], *H. hampei* [115] and *R. ferrugineus* [127,177,178,179,180]. The term “sex pheromone” is commonly defined as the chemical signals from a female to attract males of the same species for initiation of courtship or mating, whereas an “aggregation pheromone” is a male-produced attractant which draws both sexes of the same species to a calling site to increase mating likelihood [181]. The aims of these studies were to infect adult weevils with EPF by integrating the EPF with attractants as an “attract-and-infect” technique. They showed that the integration of *B. bassiana* with an aggregation pheromone component (ferrugineol or 4-methyl-5-nonanol) in the infective trap could cause high mortality to *R. ferrugineus* adults in the laboratory, but only low to moderate mortality was observed in the field [127,177,178,179,180]. Despite this apparent limitation, studies have found that the combination of *B. bassiana* with ferrugineol as part of an attract-and-infect strategy reduced infestations of *R. ferrugineus* [178,179] more effectively than the application of the insecticide chlorpyrifos alone, or the combination of chlorpyrifos with ferrugineol in an attract-and-kill system [178]. Moderate to high mortality of *C. sordidus* adults was also observed in the laboratory when *B. bassiana* was combined with an aggregation pheromone (Cosmolure^®^—sordidin or (1*S*,3*R*,5*R*,7*S*)-1-ethyl-3,5,7-trimethyl-2,8-dioxabicyclo [3.2.1] octane) [174], but again only low to moderate mortality was obtained in the field [173,175]. Similarly, Mota et al. [115] reported that only moderate mortality of *H. hampei* was observed in the auto inoculation trap containing methanol/ethanol mixture (at 1:1 *v*/*v*) and a *B. bassiana* suspension. In contrast, Yasuda [176] demonstrated high levels of control by combining *B. bassiana* conidia with sex pheromones inside a trap for controlling *C. formicarius* in the field. Although this study showed the potential for combining EPF with attractants, many others have failed to provide good control of adult weevils in the field. In the case of *H. hampei*, poor trap design [182], attractant compound selection, and inappropriate timing in relation to the emergence period of the adults [115,183] contributed to the failure of this technique. Pereira et al. [183] found that methanol/ethanol mixture is not specific to *H. hampei* and many scolytids including “false *H. hampei*”were also captured in the trap. Mota et al. [115] reported that the number of *H. hampei* captured in the trap fluctuated over 22 weeks of the experiment with the noticeable peaks of adult captures at the 5th and 7th week of the trap placement in the field. The same issues with inappropriate timing in relation to the emergence period of the adults were also raised by Sewify et al. [184] and Vacas et al. [185] in their studies on *R. ferrugineus.* Dembilio et al. [179] reported that conidia viability inside the trap significantly reduced over time, from 100% on day 1 to less than 50% at day 67, and, as a consequence, only low to moderate mortality was observed in the field. From these studies, it is obvious that to be effective combinations of EPF with attractants designed to enhance infection rates must be used when adult weevil activity is high, and utilise reliable attractants with good persistence in the field. Other key areas of work needed to optimise attract-and-infect systems include the improvement of EPF persistence in the trap, as well as enhancing the capacity of EPF conidia to adhere to the weevils.

An advantage of the attract-and-infect technique with EPF is the ability to generate local transmission between adults in the first cycle [127,173,179] and to some extent between adults and conidiated cadavers in the second cycle. The mortality of the “recipients” was around 16% for *C. sordidus* and 45% for *R. ferrugineus* when adults that had previously visited traps and served as “donors” had physical contact with them [127,173,179]. Studies showed that copulation is the main basis for disease transmission between adults via physical contact. Many studies have shown that an infected male is able to transmit EPF and subsequently cause mortality to the females, or vice versa [94,124,165,186,187]. Horizontal transmission did not just kill the female adults, but also reduced the number of eggs produced and egg viability by 44 to 68% and by 45 to 55%, respectively, for *C. formicarius* [186] and *R. ferrugineus* [125] before the females died. Interestingly, the percentage of egg viability of *R. ferrugineus* was reduced by 86–100%, after the female mated with the reproductively sterile male (gamma irradiated) carrying *B. bassiana* [188]. After adults were killed by EPF, the conidiated cadavers were also found to generate a second cycle of disease transmission. Dotaona et al. [186] reported that one conidiated cadaver could cause 63% mortality (of 10 adults) to *C. formicarius* under laboratory conditions. From these findings, infected adult weevils and conidiated cadavers have an important role in recycling and transmitting EPF within pest populations.

In addition to the capacity for horizontal transmission, EPF were also found to produce volatile organic compounds (1-octen-3-ol, 2-octen-1-ol, 3-octanol, 3-octanone) and acetic acid, which behave as repellent volatiles [189,190]. Dotaona et al. [191] found that *C. formicarius* showed avoidance behaviour toward the most virulent isolates of *M. anisopliae* when compared to controls or low virulence isolates. In contrast, Leng and Reddy [192] found that *C. formicarius* showed no avoidance behaviour toward *B. bassiana*, but avoidance was observed toward neem (a botanical insecticide), petroleum oil and the insecticide spinosad. The variation between these two studies could be explained by the work of Bojke et al. [189] who demonstrated that *M. anisopliae* was able to produce volatile organic compounds and acetic acid, whereas *B. bassiana* could not. In addition to producing potentially repellent volatiles, some EPF have been reported to have the ability to become endophytes within host plants and decrease the survivorship of weevils feeding on these hosts. Akello et al. [193,194] found that *B. bassiana* was a symbiont with banana plants and caused 53–58% mortality to *C. sordidus* adults. This reduced the population of the next generation by about 23–89%, leading to a reduction of crop damage by 42–87%. Similarly, Prabhavathi and Ghosh [87] also found that *B. bassiana* could colonise banana tissue for at least four months after dipping the corm in a conidial suspension and caused 50–70% mortality to *O. longicollis.* Date palm seedlings can also be endophytically colonised by *B. bassiana*, leading to 70–80% mortality of *R. ferrugineus* larvae when they fed on the endophytic plant in the laboratory [195].

Although numerous studies have confirmed the potential of EPF to suppress weevils in the laboratory, their variable results on horticultural crops under field conditions could lead to confusion amongst end-users or those seeking to develop and register commercial products. In order to give a better understanding of the overall potential of EPF, eleven studies have compared EPF with synthetic insecticides individually or evaluated their simultaneous use. Of the eleven papers, two showed significantly better control by EPF in comparison to synthetic insecticides alone [196,197], but another five showed the opposite result—synthetic insecticides provided superior control [43,96,192,198,199]. Only four papers have discussed synergistic interactions of EPF with sublethal doses of botanical and/or synthetic insecticides [139,200,201,202]. The combination of EPF with sublethal doses of neem and spinosad killed 100% of *C. formicarius* within 48–72 h; however, the application of the full recommended doses of either the insecticides or EPF alone took more than 72 h to kill 100% of adult weevils in the laboratory [200]. Malik et al. [202] found that the combination of *B. bassiana* with a sublethal dose of imidacloprid killed 100% of *R. ferrugineus* larvae within 20 days, whereas the same sublethal dose of imidacloprid or *B. bassiana* alone killed only 84% and 54–77% of the larvae, respectively. Again, Saleem et al. [201] and Qayyum et al. [139] found that *B. bassiana* showed synergy with a sublethal dose of nitenpyram for the control of *R. ferrugineus* adults and larvae and provided superior control to either treatment applied alone.

## 5. Effect of Fungal Entomopathogens on Weevils with Life Cycle Model 2: Larvae in the Host Plant and Pupation under the Ground

Seven screening studies have demonstrated the efficacy of *Metarhizium* spp. and *Beauveria* spp. against weevils with Model 2 habitat utilisation. In total, 28 isolates of *Metarhizium* spp. and 13 isolates of *Beauveria* spp. were used for these screening studies. Of the seven papers, five examined the effect of EPF using aqueous conidial suspensions [203,204,205,206,207] and two evaluated the use of dried conidia previously cultured on fungal media [208,209]. For the tests with aqueous conidia, adults or larvae of *Conotrachelus nenuphar* [203], *Curculio caryae* [204], *Curculio nucum* [205] and *Curculio sikkimensis* [206,207] were immersed for 8–60 s or sprayed with conidial suspensions at different concentrations. For the tests with dried conidia, *C. caryae* and *C. nenuphar* were infected by being allowed to walk or crawl for several minutes on a conidiated fungal culture [208,209]. The overall results indicated that the most virulent isolates of *B. bassiana* and *M. anisopliae* induced high mortality (>80%) to the population of weevils treated with aqueous conidia whereas 74–83% mortality of *C. caryae* and 98–99% mortality of *C. nenuphar* were obtained with the dried conidia treatments. In terms of the efficacy comparison between *B. bassiana* and *M. anisopliae*, *B. bassiana* was more active against *C. caryae* [208]; however, the opposite result was obtained with *C. sikkimensis* [207] and *C. nucum* [205].

Seventeen further papers evaluated the effect of fungal conidia applied onto or incorporated into topsoil and plant growing media (vermiculite, soybean straw) on the mortality of weevils with Model 2 habitat utilisation. These studies aimed to evaluate EPF for control of either larvae below the ground, or adult weevils moving on the ground or on plant growing media. Nine studies were conducted in the laboratory and the remaining studies assessed efficacy under field conditions with the most virulent isolates and commercial strains/products (*M. brunneum* strain F52, *B. bassiana* strain GHA—Mycotrol^®^, Botanigard^®^, *B. bassiana* strain ATCC 74040—Naturalis^®^). In the laboratory, EPF were sprayed or applied as a drench onto the soil or plant growing media and left for 1–24 h before the introduction of larvae or adults onto the sprayed surface. Moderate (60–80%) to high (>80%) mortality of weevils such as *C. nenuphar*, *C. caryae*, *C. elephas* and *C. nucum* was obtained after treatment with virulent isolates [209,210,211,212], Mycotrol^®^ and Naturalis^®^ [213,214,215] but only low (<60%) to moderate (60–80%) levels of control were achieved when adult weevils or larvae (e.g., *C. caryae*) were introduced four days after EPF application [208,216]. Low to moderate mortality of *C. caryae* [204,217,218], *C. sikkimensis* [206,207], *C. nucum* [219] and *C. nenuphar* [220] was also achieved in the field after the application of virulent EPF isolates or commercial products (Beaupro^®^, Metapro^®^ and Botanigard^®^), suggesting that the weevils moved onto the ground several days after fungal application [208,216]. To mitigate poor infectivity of EPF in the field, Shapiro-Ilan and Brown [215] suggested that using earthworms (*Lumbricus terrestris*) as phoretic hosts for *B. bassiana* in the soil could improve fungal infectivity in comparison to the more traditional applications of EPF directly to the topsoil. Other studies found challenges still remain that complicate efforts to control pecan weevils successfully with EPF. Shapiro-Ilan et al. [221] found that high infectivity of EPF did not persist in the field for longer than one week after application. The number of conidia recovered from field soil declined significantly one week after application, from around 6.5 × 10^3^ CFU/g of soil at day 1 to around 3 × 10^3^ CFU/g of soil at day 8 in a 2009 trial and from 9 × 10^2^ CFU/g of soil at day 1 to 1 × 10^2^ CFU/g of soil at day 8 in a trial conducted during 2010 [218]. The number of conidia recovered from the soil continued to drop to almost zero by day 29 in both years [218]. The authors suggested that conidial densities declined rapidly because there was no mulch or cover crop to provide protection from UV radiation penetrating the crop canopy and contribute towards stabilising topsoil temperature and humidity [218]. Shapiro-Ilan and Mizell [222] also found that the pupal cell of *C. caryae* had antimicrobial properties that had the potential to inhibit penetration and infection by the fungi. Long gaps between fungal application and weevil activity, poor persistence and uneven distribution of EPF in the field, and antimicrobial properties of the pupal cell are all factors that can contribute to the reduced efficacy of fungal applications in the field.

Only four studies have evaluated the efficacy of EPF on weevils of pecan in the field. Spray applications targeting *C. caryae* adults on the plant showed that the most virulent isolates of *M. anisopliae* and Botanigard^®^ (containing *B. bassiana* strain GHA) caused moderate (60–80%) to high (>80%) levels of mortality to *C. caryae* whilst *M. brunneum* strain F52 caused low (<60%) to moderate (60–80%) mortality [217,223,224,225]. Interestingly, spray applications of EPF on the plant caused slightly higher mortality of *C. caryae* than the application of EPF on the ground [217,223,225]. Although moderate to high mortality of *C. caryae* was achieved, the authors noted that economic control was not achieved as the EPF required weeks to kill the weevils, during which time the weevils continued to cause damage to the crop. Although mortality may have been delayed, these infected adults may play an important role in the horizontal transmission of EPF, providing more effective control in the longer term. In the case of *C. caryae*, infected males or females were able to transmit EPF via contact during mating, leading to 50% mortality in their partners [216].

As fungal applications to the ground and foliage have not achieved the optimum level of control, EPF have been tested and integrated with other components of IPM programs including chemicals and other biological control agents. Combinations of EPF with EPNs (*Heterorhabditis bacteriophora*, *Steinernema carpocapsae*, *S. feltiae*) induced moderate (60–80%) to high (>80%) levels of mortality of *C. caryae*, *C. elephas* and *C. nucum* larvae [210,226,227]. These combinations (EPF + EPNs) did not, however, provide any significant advantages over individual treatments (EPF or EPNs alone), which also caused good levels of mortality in the targeted weevils. This was confirmed by Shapiro-Ilan et al. [228] who found that EPF + EPNs did not result in mortality of *C. caryae* higher than that caused by EPF or EPNs alone, and by Batalla-Carrera et al. [210] and Asan et al. [227] who reported that the mortality of *C. elephas* and *C. nucum* caused by EPF + EPNs and EPF alone did not differ. It is difficult to draw direct comparisons between EPF and EPNs because their mode of action and their effective concentrations are different. Studies show that when EPF or EPNs are applied on the topsoil before introducing weevil larvae, the EPNs provide better control of both *C. caryae* [226] and *C. nenuphar* [220] compared to EPF. However, when larvae are immersed in the fungal suspension and compared with EPNs (applied on the topsoil), the mortality of both *C. elephas* [227] and *C. nenuphar* [203] caused by EPF was always higher than that caused by EPNs. These studies suggest that in many cases EPNs are likely to provide better control of weevils active below the soil surface than EPF. This is discussed further in the following sections.

Combinations of EPF with synthetic insecticides have shown their potential synergy, with 100% control of weevils including *C. caryae* [229] and *C. psidii* [230]. When *B. bassiana* was applied together with a sublethal dose of imidacloprid to *C. psidii*, 100% mortality of adults was recorded; however, the application of *B. bassiana* alone killed only 62% of weevils and the sublethal dose of imidacloprid alone did not kill any [230]. The sublethal dose of imidacloprid increased the vulnerability of weevils to EPF, presumably by diverting metabolic activity to insecticide detoxification and thereby reducing the insect’s capacity to resist fungal infection. The authors also observed that the sublethal dose of insecticide had a substantial impact on the insect’s grooming behaviour [230]. The impact of sublethal doses of insecticides on the grooming behaviour of weevils and its implications for EPF efficacy will be discussed further in the section on weevils with Model 3 habitat utilisation.

## 6. Effect of Fungal Entomopathogens on Weevils with Life Cycle Model 3: Larvae and Pupae under the Ground

Twelve screening studies have demonstrated the efficacy of *Metarhizium* spp. and *Beauveria* spp. on four weevils (*Aegorhinus superciliosus*, *Diaprepes abbreviatus*, *Otiorhynchus sulcatus* and *Phlyctinus callosus*) with Model 3 habitat utilisation. In total, 56 isolates of *Metarhizium* spp., 29 isolates of *Beauveria* spp. and 7 commercial strains/products were used for these screening studies. All studies examined the effects of EPF using aqueous conidial suspensions. Larvae or adults of *A. superciliosus* [231], *D. abbreviatus* [232], *O. sulcatus* [233,234,235,236,237,238,239,240,241] and *P. callosus* [242] were immersed for 10–60 s, exposed to a topical application, or sprayed with conidial suspensions at different concentrations. The comparison of *Metarhizium* spp. and *Beauveria* spp. showed that *Metarhizium* spp. often performed better than *Beauveria* spp. against *O. sulcatus* [235,236,237], although some studies found that both fungi were equally effective in killing *O. sulcatus* and *P. callosus* [238,240,242]. In general, the majority of the most virulent isolates or commercial strains/products and/or the highest concentration used in each study caused moderate (60–80%) to high (>80%) level of mortality to the target weevils.

At least 17 papers have evaluated the effect of application or incorporation of fungal conidia into or onto topsoil or plant growing media (bark-based potting medium, peat-based media, spent mushroom compost, peat moss, peat compost, turkey grit) on weevil mortality. These studies aimed to use EPF to control target insects as below-ground larvae, or as adults dispersing across the soil surface towards a plant host. Conidia were sprayed onto or drenched into the soil or plant growing media before introducing larvae or adults onto the treated surface under laboratory conditions. Moderate (60–80%) to high (>80%) mortality of the weevils was observed [236,243,244,245,246,247,248,249,250,251,252,253,254]. The commercial strains/products Met52^®^ and Naturalis^®^ showed strong and consistent control of weevils and caused high (>80%) levels of mortality [248,250,254]. However, application of Met52^®^ as a topsoil drench in the field provided only low mortality of *O. sulcatus* larvae [255]. Moderate to high mortality of the weevils resulted when conidiated rice was applied directly on the topsoil, providing an infective layer for controlling the larvae of this species [256,257] suggesting this approach may provide sufficient conidia on the topsoil to adhere to and cause mortality to *O. sulcatus*. The mortality of *O. sulcatus* was high in the first week after EPF were incorporated with a bark-based potting medium, but mortality decreased to moderate (60–80%) levels after 77 days [249] and zero after 1 year [258]. This is likely the result of conidia degradation, as observed by Bruck [248] who reported that the number of conidia recovered from peat-based and bark-based potting media reduced gradually; from around 1 × 10^6.5^ CFU/g dry potting media at week 2 to around 1 × 10^5.5^ CFU/g dry potting media at week 48. Shapiro-Ilan et al. [218,225] reported the number of conidia recovered from topsoil dropped to almost zero 7 weeks after an EPF application in the field. To minimise rapid conidial degradation in the field, several studies have recommended that EPF should be incorporated with pasteurised potting media (such as peat-based and bark-based potting media) for at least one week before use [248,250]. This allows the EPF to adjust to the media and grow in controlled conditions with good moisture levels and nutrients before being used in the field.

Pope et al. [238] found that the use of *M. brunneum* (F52) conidial powder in Roguard refuges (black plastic crawling insect stations) provided at least 93% control of *O. sulcatus* after 28 days. Deployment of *B. bassiana* (GHA strain) under the same conditions provided only 27–67% weevil mortality. Other studies have also demonstrated that *Metarhizium* spp. perform better than *Beauveria* spp. against *O. sulcatus* [235,236,237]. Baits or attractants are not essential in Roguard refuges for *O. sulcatus* control since adults of this weevil are nocturnal and move inside the station during the day to avoid exposure to sunlight [64]. The recent development of lures ((Z)-2-pentenol + methyl eugenol) for *O. sulcatus* [259] has, however, improved the attractiveness of the refuges, and deploying the lures with an EPF formulated in linseed oil within the refuges has provided very effective control of *O. sulcatus* in the field [260].

The behaviour of weevils in response to volatiles produced by EPF has also been explored. Rondot and Reineke [261] found that *O. sulcatus* has the ability to detect EPF and avoids the commercial product Naturalis^®^ and Kepler and Bruck [262] showed that whilst *O. sulcatus* does not avoid *M. brunneum* (strain F52), it does avoid the insecticide bifenthrin. Although *O. sulcatus* showed avoidance behaviour in response to Naturalis^®^ [261], this could be a response to additives in the commercial product rather than to *B. bassiana* itself. A recent study found that *B. bassiana* does not produce repellent volatiles [189].

Entomopathogenic fungi synergism with other entomopathogens and insecticides has also been studied in relation to weevils with Model 3 habitat utilisation. Fungal entomopathogens were applied alone or together with EPNs (*Heterorhabditis bacteriophora*, *Steinernema kraussei* and *S. feltiae*) on the topsoil and plant growing media (peat-based media) before introduction of *O. sulcatus* larvae. High mortality of larvae was obtained when *Metarhizium* spp. were combined with EPNs in the laboratory, glasshouse and to an extent in the field [263,264,265]. *Metarhizium* spp. seemed to dominate the detrimental effects on the larvae; when used individually, the mortality of larvae caused by *Metarhizium* spp. alone was 40–88% in the glasshouse and 88–94% in the field, and the mortality of the larvae caused by the EPNs was 30–69% in the glasshouse and 20–75% in the field [263,264,265]. High levels of mortality were also found when EPF were applied together with synthetic insecticides against weevils in this group. More than 90% mortality of *D. abbreviatus* and *O. sulcatus* occurred when EPF were applied together with sublethal doses of imidacloprid, fipronil or neem either directly or via peat-based plant media (a combination of peat, bark, coir and compost) before introduction of the larvae [266,267,268,269,270]. However, the application of either EPF or sublethal doses of insecticides alone caused only low (<60%) to moderate (60–80%) mortality of the target weevils. The lower mortality of the larvae treated with EPF alone is attributed to below-ground movement of the larvae leading to the passive removal of conidia as the larvae moved against soil particles, or active removal associated with grooming behaviour [266,267,268]. The removal of fungal conidia during grooming has also been observed in adults [271]. A sublethal dose of imidacloprid led to reduced or temporary loss of mobility by the larvae, which were then unable to remove fungal conidia from their cuticle [266,267,268].

## 7. Integration of Fungal Entomopathogens in the Integrated Pest Management Programs and Future Research Directions

Entomopathogenic fungi have shown potential to control many weevil species associated with horticultural crops under laboratory conditions, but wide variations in weevil mortality are commonly seen across different fungal species, isolates and strains. In some cases, the fungal strains which were isolated from particular weevils have shown limited capacity to control that species (e.g., coffee berry borer, *H. hampei* [108,112], banana weevil, *C. sordidus* [96,97] and red palm weevil, *R. ferrugineus* [128]). In contrast, other studies have shown that strains of EPF which naturally infect target weevils work better against those species than strains baited from the soil, commercial strains, or commercial formulated products [94,95,115,132]. As there are no consistent patterns in these studies, it is most appropriate to use a registered commercial strain as a reference strain and compare this with any newly isolated strains in screening studies. This will provide more useful baseline data on the relative virulence of new strains, which should be assessed on their potential to provide improvements relative to existing commercial products rather than relative to other, often randomly selected experimental isolates.

Although there were only four studies on the response of weevils to *B. bassiana* deployed as fungal endophytes in plants (all life cycle habitat utilisation Model 1 species) [87,193,194,195], the establishment of endophytic plants is an effective preventive tactic, and a practical solution for managing weevils of horticultural crops. In addition to causing mortality of the weevils, endophytic plants may have less damage and yield loss, as the fungi that colonise the host plant [193,194] probably produce insecticidal metabolites which may improve the resistance of the host plant to attack [16,272,273]. The establishment of fungal endophytes within annual crops has often been noted [272,273]; however, no studies have been performed on perennial crops that have extended beyond the seedling stage. Studies on seedlings have included those on pecan [274], cacao [275], coffee [276], and those on isolated plant parts [277,278,279]. Further research on the protection provided by fungal endophytes in mature perennial crops is needed, with a focus on the persistence of endophyte activity in the plant and correlating this to effects on pest populations. The methodology for confirming endophytic activity is crucial for separating the effects of endophytes from those associated with epiphytic fungi [16].

The application of EPF incorporated into plant growing media around and below crops to produce a “contamination layer” or “infective zone” has been shown to provide long-term control of adult and larval weevils. Incorporating EPF with pasteurised organic fertilizers, compost or growing media [248,249,250] in combination with zero-tillage [280,281] may improve not just the abundance, but also the persistence of EPF in the soil. This approach may help compensate for the problems associated with the limited durability and infectivity of EPF in horticultural crops. Although EPF can in some cases persist in the soil for long periods [282] (up to 15 years in exceptional cases [283]), a single application of EPF on the topsoil may have only short-term benefits for pest management, as the fungal density usually decreases gradually after application. This theory is supported by many studies [237,284,285]; however, despite its transient nature, the application of compost, organic fertilizer or plant growing media colonised by EPF around crops probably represents the best EPF-based technique to control weevils that have a predominantly subterranean pattern of habitat utilisation. More importantly, this solution is suitable for organic growers who are required to use only compost or organic fertilizer on their crops.

Identification of effective attractants for additional weevil species should allow further development of attract-and-infect or attract-and-kill techniques utilising the most virulent strains of EPF, helping to minimise application and management costs. Integration of EPF (particularly *B. bassiana* and *M. brunneum*) with an attractant was far more effective than combining the attractant with insecticides [178] because weevils were able to detect and avoid many insecticides (e.g., bifenthrin [262], spinosad, neem, petroleum oil [192]), whereas *B. bassiana* and *M. brunneum* do not produce repellent compounds [189] and are consequently suitable to integrate with attract-and-kill systems. The use of adhesive carriers for conidia, such as electrostatically charged powders, will also help to improve the success of attract-and-infect and attract-and-kill techniques. This approach has been successfully integrated with both EPF and synthetic pesticides to control stored product pests [286,287,288], varroa mites [289], and mosquitoes [290]. Carriers improve the ability of conidia to transfer more easily to the insect and in sufficient numbers to cause mortality, both directly or by subsequent transfer to other individuals. Although attract-and-infect and attract-and-kill systems are good in theory, these techniques may not be applicable to all species, since attractants may be difficult to identify and synthesise, and some species may not utilise pheromones for aggregation or mate location to begin with. Where pheromones or other attractants are known, however, their integration with EPF in these sorts of systems represents a great opportunity for reducing dependence on synthetic insecticides.

**Figure 2:**
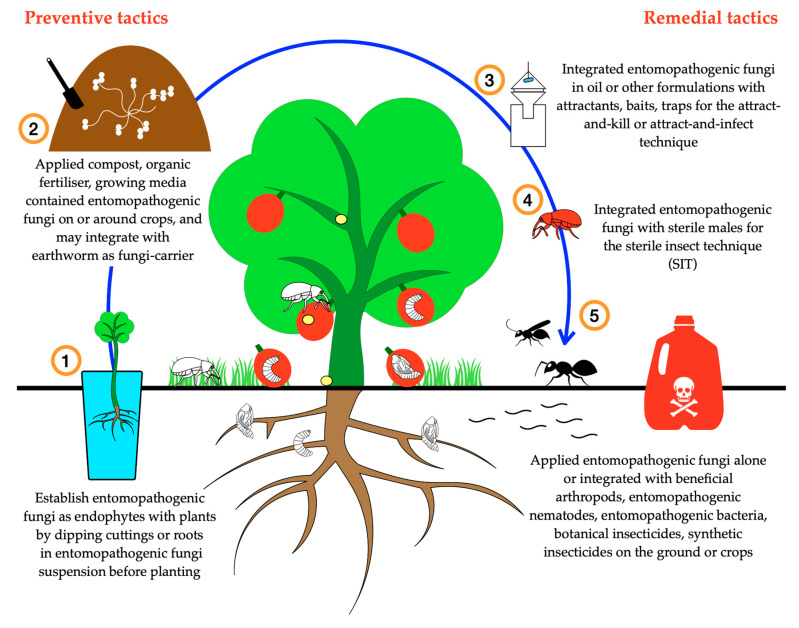
Conceptual illustration of potential uses of entomopathogenic fungi in IPM programs for managing weevils of horticultural crops.

One of the most interesting techniques for utilising EPF involves the horizontal transmission of conidia from male weevils sterilised using ionising radiation (Figure 2). Of the journal papers examined in this review, only one paper tested this technique. Significant control of the red palm weevil *R. ferrugineus* was reported [188] and the sexual competitiveness of sterile males was not reduced by sterilisation when compared to non-sterile males [291]. The combination of the sterile insect technique (SIT) with EPF has also been tested on fruit flies including Mexican fruit fly (*Anastrepha ludens*) [21], Mediterranean fruit fly (*Ceratitis capitata*) [19], melon fly (*Bactrocera cucurbitae*) [20], and peach fruit fly (*Bactrocera zonata*) [292]. The sterile insect technique alone or used in combination with EPF has shown potential for safe and selective pest control, but, since sterilisation may have a negative impact on sexual competitiveness [21], further research on optimising sterilisation procedures is needed for each species being targeted.

Although commercial strains of EPF have been regularly used in the field, only moderate levels of control have been obtained [217,218,220,223,225]. This is largely attributable to the negative impact of unfavourable weather conditions [293]. There have been many efforts to improve the formulation of EPF to withstand unfavourable environmental conditions including high temperatures and UV radiation [294,295], but recent efforts have been focussed on finding weather tolerant strains [296,297] and understanding and improving the tolerance of the fungi themselves to heat and sunlight [298,299].

To the best of our knowledge, EPNs and *B. thuringiensis* are the only other biological control agents to be experimentally integrated with EPF. As the mode of action of *B. thuringiensis* is by ingestion, it is suitable for integration with EPF for application to aerial parts of the host plant rather than to the topsoil, and this represents a useful approach for controlling weevil adults feeding on the crops. Several studies have shown the potential of *B. thuringiensis* toxins for controlling weevils of horticultural crops including *C. puncticollis*, *C. brunneus*, and *D. abbreviatus* [300,301] and stem from the findings of Malik et al. [170] that *B. thuringiensis* is suitable to integrate with EPF for controlling other weevil species. Entomopathogenic nematodes are suitable to integrate with EPF for application to the ground rather than to the trunk or foliage of the plant, and this represents a useful approach for controlling larval weevils with life cycle habitat utilisation Models 2 and 3 (Figure 2). Almost all combinations of EPF with EPNs have proven to be positive and caused significant mortality to the target weevils (e.g., *R. ferrugineus*, *C. nenuphar*, *C. caryae*, *C. elephas*, *C. nucum* and *O. sulcatus*) which they were tested against. In addition, the rotational application of EPF and EPNs at two-week intervals was found to be effective against weevils, especially by Anbesse et al. [302] who also found that three-week intervals were effective. The simultaneous or sequential applications of EPNs and EPF on the soil surface or onto plant growing media produces a “contamination layer” or “infective zone” that brings larval weevils and the biological control agents into close contact, facilitating infection. Entomopathogenic nematodes seem to have an advantage for controlling larvae with Model 2 and 3 habitat utilisation patterns, as they are active entomopathogens, able to move freely in the soil and ambush their hosts which are active below the soil surface. In contrast, EPF are passive entomopathogens and insect infection relies on movement of the host to provide contact with the conidia, particularly when the larvae exit from plant tissues and move into the soil. Achieving EPF infection in weevil larvae living more than a few centimetres below the soil surface is particularly difficult and highlights the need for control methodologies to be chosen based on a thorough knowledge of pest biology and the persistence of entomopathogens in the rhizosphere.

Several studies have focused on the direct effect of EPF on predators of *A. signatus* (e.g., the generalist predatory bug *Anthocoris nemorum* [303]), *C. sordidus* and *R. ferrugineus* (e.g., the predatory earwig *Euborellia annulipes* [304]), *D. abbreviatus* (e.g., Asian lady beetle *Harmonia axyridis* and the generalist predatory lady beetle *Olla v-nigrum* [305,306,307]) and parasitoids of *H. hampei* (e.g., the bethylid ectoparasitoid *Prorops nasuta* [308], egg parasitoid *Trichogramma pretiosum* [309,310,311], eulophid endoparasitoid *Phymastichus coffea* [312] and bethylid ectoparasitoid *Cephalonomia stephanoderis* [313]). These studies indicate that the integration of EPF with predators and parasitoids should be feasible, but EPF should be applied at different times relative to any supplementary releases of beneficial insect species. For example, to effectively integrate EPF with *T. pretiosum* (an egg parasitoid), studies suggest that *T. pretiosum* should be released around three days prior to the application of EPF on crops and the second application of EPF should be delayed for a minimum of seven days after the first application. This timing ensures that when the EPF is applied the majority of parasitoids are developing within host eggs, rather than being exposed to the EPF as adults, since the development of *T. pretiosum* from egg to adult takes around one week [309,310]. Application of EPF to host eggs already parasitised by *T. pretiosum* did not have any negative impact on subsequent emergence of the parasitoid [309,310]. In contrast, the application of EPF to crops before releasing adult *T. pretiosum* may lead to *T. pretiosum* avoiding oviposition into host eggs already infected by the EPF [309,310]. The generalist predatory bug *A. nemorum* is known to avoid prey that are already infected by EPF and the avoidance behaviour was more pronounced towards conidiated cadavers. In addition, adults also avoided laying eggs on the plants that had already been treated with EPF [303]. Although these examples recommend releasing parasitoids and predators before the application of EPF, the optimum timing of EPF applications relative to releases of predators or parasitoids is likely to be specific to each combination of pest, EPF and beneficial species involved, and further studies in this area are required.

Some combinations of EPF with sublethal doses of botanical and synthetic insecticides have been shown to be synergistic and this interaction can also provide an effective solution for the management of weevils on horticultural crops. Combination treatments may work better than applications of either EPF or insecticide alone because the insecticide may disrupt insect grooming behavior that would otherwise lead to the removal of conidia before their germination [266,267,268,271]. Vulnerability to fungal infection in the insects may also be increased as a consequence of stress caused by insecticide exposure [230]. Although these combinations often show positive results, the use of sublethal insecticide doses may not be possible in field applications due to regulatory requirements designed to specifically combat resistance to standalone insecticide treatments caused by underdosing. In addition, not all synthetic insecticides are synergistic with EPF. In some studies, synthetic insecticides were toxic to EPF in tank mixes [314] and combined applications cannot be recommended. Adverse interactions may be a consequence of either the active ingredient or formulation additives being toxic to the entomopathogens [314]. Modifying the insecticide formulation may help avoid this problem; however, if the active ingredient is toxic to the fungus, the only viable option may be to separate the applications in time, and this requirement may be particularly significant with regard to the potential development of pesticide resistance.

## 8. Conclusions

In conclusion, entomopathogenic fungi are amongst the most promising biological control agents for use against weevils affecting horticultural crops. Based on the 175 peer-reviewed studies we examined, it is clear that the success of weevil IPM programs relies on having detailed knowledge of the biology of the species involved. Three groups of life cycles based on the weevils’ developmental habitats have been recognised in this study and the susceptibility of each group to EPF has been reviewed in the context of their possible pathways of exposure. The integration of EPF into both preventive and remedial aspects of IPM programs using the methods discussed in this review and targeting developmental stages in habitats that make them most vulnerable to EPF infection will help reduce dependence on synthetic insecticides for weevil management in many of the world’s major horticultural crops.

## Supplementary Materials

The following are available online at https://www.mdpi.com/2075-4450/11/10/659/s1. Figure S1: Flow diagram illustrating the selection process for publications include in this review. Figure S2: (A) The number of published studies using fungal entomopathogens on each weevil species affecting horticultural crops and included in this review, and (B) published studies using fungal entomopathogens for controlling weevils affecting horticultural crops and included in this review from 1973 to 2020.
