# Supplementary material for: Integration of Entomopathogenic Fungi into IPM Programs: Studies Involving Weevils (Coleoptera: Curculionoidea) Affecting Horticultural Crops
Source: Insects. 2020 Sep 25;11(10):659. doi: 10.3390/insects11100659 (PMC7599691; doi:10.3390/insects11100659)
Supplement: Supplementary file 1 [file insects-11-00659-s001.pdf]

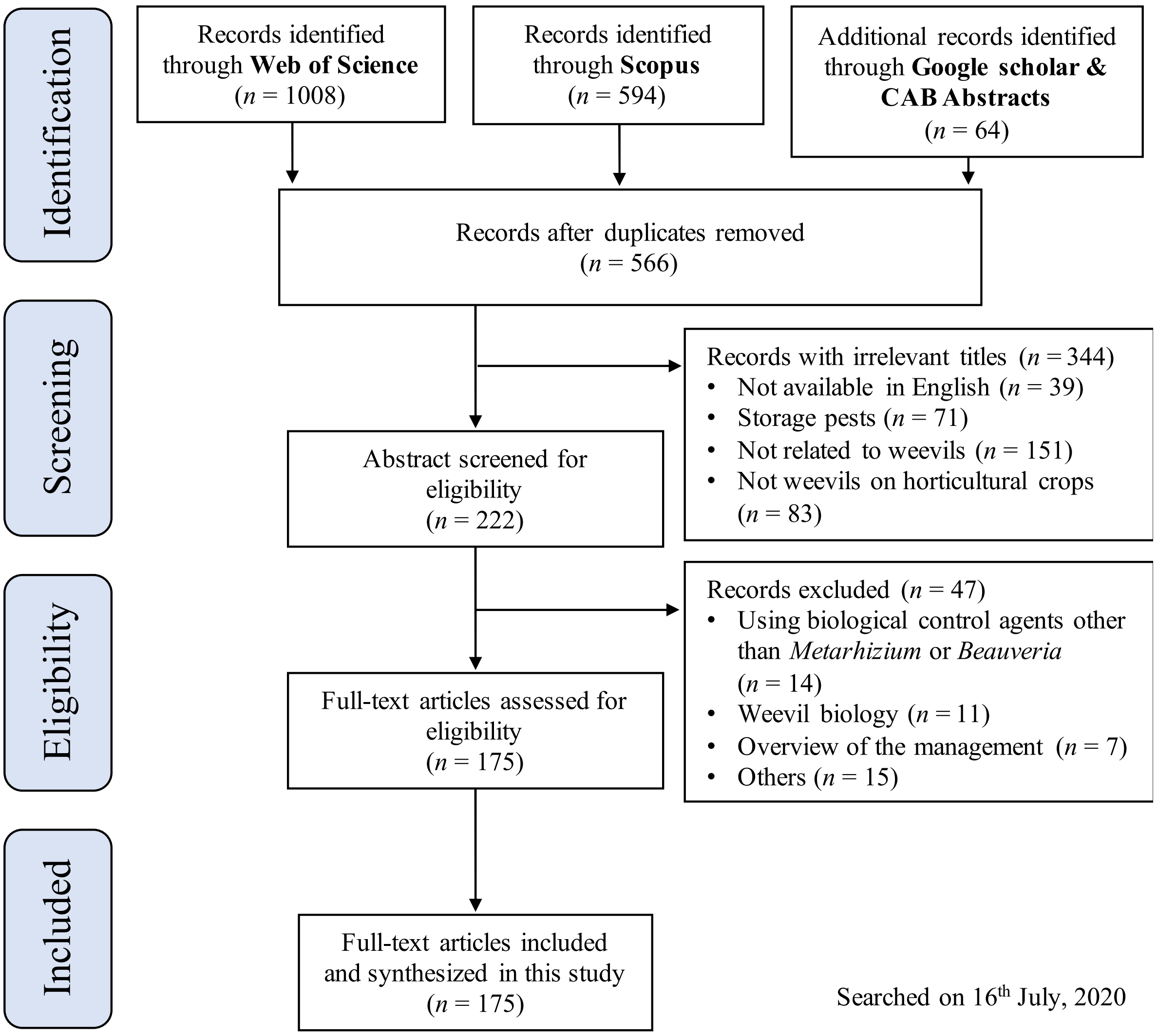

Figure S1: Flow diagram illustrating the selection process for publications include in this review

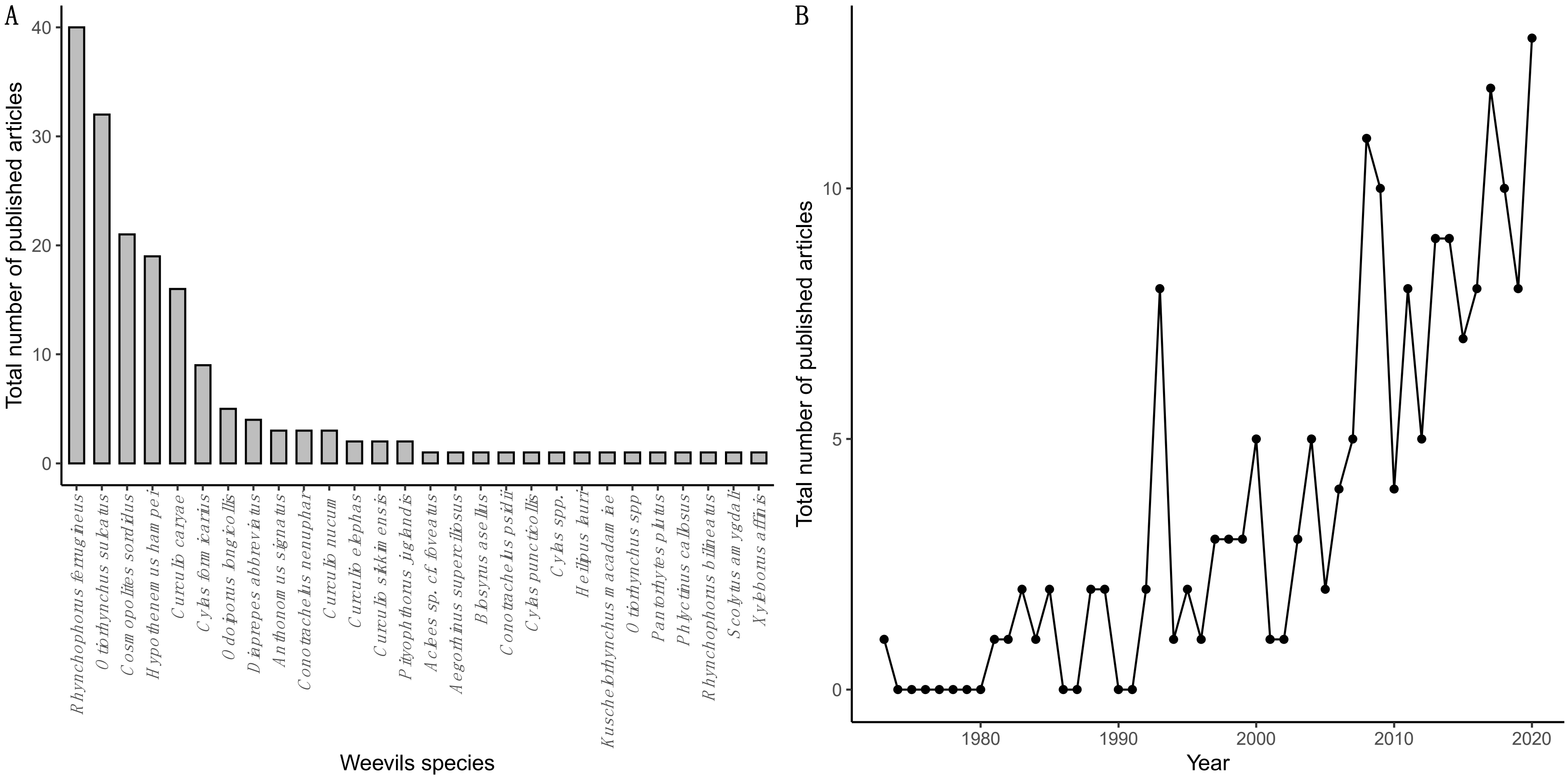

Figure S2: (A) The number of published studies using fungal entomopathogens on each weevil species affecting horticultural crops and included in this review, and (B) published studies using fungal entomopathogens for controlling weevils affecting horticultural crops and included in this review from 1973 to 2020. Last accessed on 16th July, 2020.
